# Effects of infection control measures towards preventing SARS-CoV-2 outbreaks in a German choir boarding school from March 2020 to April 2022

**DOI:** 10.3389/fped.2023.1215678

**Published:** 2023-08-08

**Authors:** Benedikt M. J. Lampl, Patricia Schöberl, Noah Atzenbeck, Michael Erdl, Nepomuk Dillitzer, Jakov Wallbrecher, Marcus Weigl, Michael Sauer, Parastoo Kheiroddin, Jakob Niggel, Richard Mauerer, Andreas Ambrosch, Michael Kabesch

**Affiliations:** ^1^ Division of Infection Control and Prevention, Regensburg Department of Public Health, Regensburg, Germany; ^2^ Department of Epidemiology and Preventive Medicine, Faculty of Medicine, University of Regensburg, Regensburg, Germany; ^3^ University Children's Hospital Regensburg (KUNO) at the Hospital St. Hedwig of the Order of St. John, University of Regensburg, Regensburg, Germany; ^4^ Regensburger Domspatzen, Regensburg, Germany; ^5^ MaganaMed GmbH, Regensburg, Germany; ^6^ Synlab Weiden GmbH, Weiden, Germany; ^7^ Institute of Laboratory Medicine, Microbiology and Hygiene, Hospital of the Order of St. John, Regensburg, Germany

**Keywords:** SARS-CoV-2, outbreaks, rRT-PCR pool-testing, bundle strategy, choir singing

## Abstract

**Background:**

Singing in a choir was associated with larger outbreaks in the beginning of the SARS-CoV-2 pandemic.

**Materials and methods:**

We report on the effect and acceptance of various infection control measures on the occurrence of SARS-CoV-2 infections in the world famous Domspatzen boys' choir from March 2020 to April 2022.

**Results:**

In addition to basic general hygiene measures, systematic rRT-PCR testing and scientifically approved concepts of distancing during singing were applied. While single infections of choir members could not be avoided, singing-related outbreaks were not observed. Until the Omicron variant emerged, potential transmission of SARS-CoV-2 in the school was limited to only one case. Incidences at the school were never higher than in the comparable general population until then. While the impact of the pandemic on daily life and singing was rated as severe, especially by staff members, most students agreed with the usefulness of protection measures and rated them as acceptable. Students viewed regular testing as the most important tool to increase safety in the school.

**Discussion:**

A bundle of infection control measures including regular testing can prevent outbreaks of SARS-CoV-2 even in the setting of choir singing. Measures are acceptable for choir members if they allow to continue with singing and performing.

## 1. Introduction

Singing in a choir was associated with larger outbreaks of severe acute respiratory syndrome coronavirus-2 (SARS-CoV-2) infections in the beginning of the pandemic, receiving great media attention (1, 2). Outbreaks associated with choir activities were reported from different countries and airborne transmission of SARS-CoV-2 is well characterized in the literature and attributed to the extensive production of infectious aerosols during singing (3–5). Thus, choirs and choir schools around the world were closed down or forced to introduce elaborated safety measures to prevent further outbreaks (6, 7). In Germany, schools and childcare centers in general, including choir schools, were closed very early during the pandemic and only hesitantly reopened as one of the last areas of life (8).

Not only closing schools but also suspending choir activities was a dramatic step for choir schools such as the Regensburg Domspatzen, a world-famous boys' choir dating back to the year 975. Therefore, numerous measures were installed and followed scientifically in that school to prevent outbreaks of SARS-CoV-2, including distancing during singing and regular PCR testing concepts. The STACADO project (Study to Avoid Out-breaks of Coronavirus At the DOmspatzen), was initiated to address the question how the particular risk of SARS-CoV-2 transmission could be minimized while maintaining regular choir activities. To that end, gargle pool real time polymerase chain reaction (rtPCR) tests were initiated and explored in the STACADO project and institutionalized in the WICOVIR project (Where Is the COrona VIrus?) (9). Before starting rRT-PCR pool testing at the beginning of the schoolyear 2020/2021, antibody responses to SARS-CoV-2 were assessed in members of the Domspatzen school (students and staff) in the COKIBA project (COronavirus Antibodies in KIds in BAvaria) (10). Here we report on the outcomes (1) of outbreak avoidance and case numbers among Domspatzen singers in comparison to the general population and (2) the acceptance of these measures by Domspatzen students and staff.

## 2. Materials and methods

### 2.1. Study design, study population and data collection

Students of the Regensburg Domspatzen boarding school were aged 10 to 21 when they participated between September 2020 and April 2022. The students/singers were allowed to leave the boarding school for home visits to their families. Pupils were allowed to move freely in public whenever this was allowed according to general rules and no lockdown was imposed. Visitors were also welcome in the house, but only under the respective regulations: registration at the gate, proof of a negative SARS-CoV-2 test, compliance with hygiene regulations, mouth and nose cover, distance, etc. in the house. It was clear to all the singers that they had to be conscious and cautious in public at all times and places if they didn't want to jeopardize singing and performing, potentially putting indirect pressure on the children. This was a major reason why we performed a survey on the potential burden of these measures on the choir members.

In the school year 2020/2021, 282 students attended school, and 265 in the school year 2021/2022, while 138 staff members worked at the school during this time. While general hygiene measures were mandatory, participation in all study procedures was voluntary. Informed consent was obtained from parents, students and staff who were willing to participate in the studies STACADO, WICOVIR and COKIBA. Briefly, STACADO explored the feasibility of gargle pool rRT-PCR testing in the school in a pilot project exclusively to the Domspatzen choir school setting and assessed effectiveness of testing and overall preventive measures. WICOVIR (Where Is the COrona VIRus?) initiated gargle pool rRT-PCR testing in regional schools in general based on STACADO experience (9, 11), and COKIBA assessed the antibody response to SARS-CoV-2 in children in Bavaria from as early as spring 2020 (10). Details about the respective study procedures and questionnaires were made available through study websites (https://we-care.de/stacado). All studies were approved by the Ethics Committee of the University of Regensburg STACADO: file-number: 20-1953-101, WICOVIR: file-number 21-2240-101, COKIBA: file-number 20-1865-101. All methods were carried out in accordance with relevant guidelines and regulations. The images of Domspatzen singers used for this publication have officially been approved by Domspatzen for public use.

Data collection in the STACADO project was based on the secure qnome.eu browser-based software (MaganaMed GmbH, Regensburg). Study participants were contacted via the school only in the course of the study. Teachers and school personnel were trained in all study procedures before the study began. Participants were provided with an individual code upon study participation. All laboratory results were linked to that code only known to the participant. With the code, only the participant was able to view individual results from SARS-CoV-2 testing online in real time as soon as test results were available. The laboratory performing the tests and the study personnel only had knowledge of the study code. In the COKIBA project (10), only study participants were able to view their individual SARS-CoV-2 antibody values with their unique and individual study code in the secure qnome.eu browser-based software (MaganaMed GmbH, Regensburg). In the WICOVIR project (9), which assessed rRT-PCR pool information only, no personal or medical data of the participants were collected. Only the responsible staff of Domspatzen boarding school had information who was part of a specific pool. The respective records of the school were only used for resolving positive pools and deleted within 24 h. A browser-based software tool from MaganaMed GmbH, Regensburg, was employed for pool test logistics using pseudonymized pool IDs and alphanumeric sample IDs only as previously described (9, 11). Individuals could be identified exclusively by the school and the diagnostic lab upon request the public health authorities, respectively. Also the anonymous survey on acceptance of measures at the Domspatzen boarding school was implemented using the qnome.eu browser-based software.

### 2.2. General and specific hygienic measures

**Figure 1:**
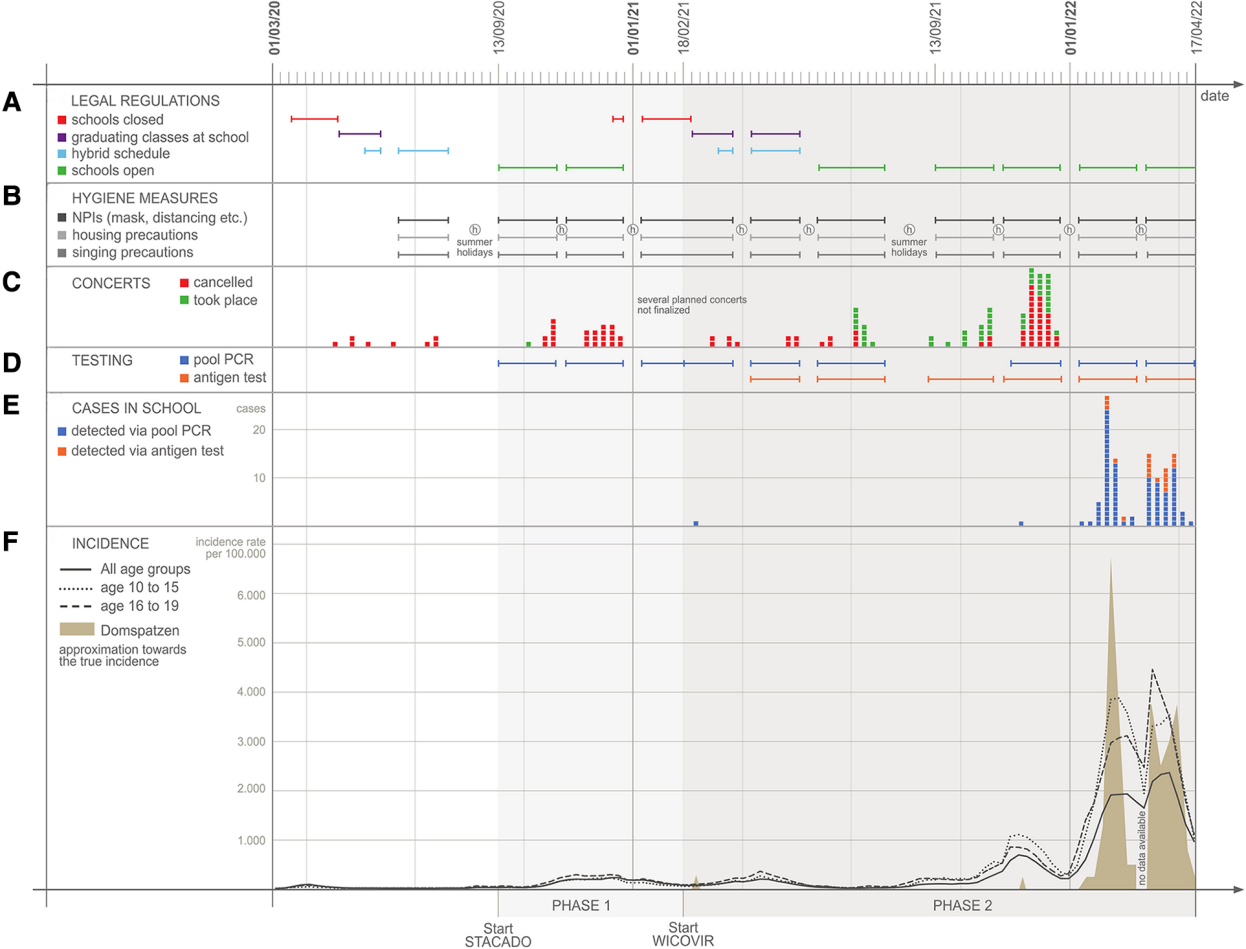
Time course of legal regulation (Panel **A**) infection control measures (**B**), concerts (**C**), testing procedures (**D**) and SARS-CoV-2 cases in the domspatzen boarding school (**E**) and in the general population (**F**) during the first 2 years of the pandemic.

During the study period, all general hygiene and protective measures (non-pharmaceutical interventions, NPI) as implemented by the public authorities were observed (Supplementary Table S1): distancing, coughing- and hand-hygiene, wearing of a medical mask or filtering face piece 2 (FFP2) respirator. As a boarding school, further precautions were taken for housing. The number of students in dormitories was reduced to two, meals were taken in set groups and activities such as sport and music instrument practice had to be reduced or stopped according to official regulations. Furthermore, the Regensburg Domspatzen applied an elaborate hygiene concept for singing practice from the beginning while most concerts had to be canceled, see Figure 1 Panel C. This included individual singing practice instead of choir practice, choir practice in set groups with a limited num-ber of participants and distancing scheme during singing, based on earlier studies on aerosol development and distribution in choirs (12). Over time, the distance between singers changed, ranging from 5 meters in singing direction at the beginning of the lockdown to 1 meter after implementing a closely-knit testing regime in September 2021. As air filters were not feasible during the early phase of the pandemic, regular cross ventilation within short time periods was performed instead (Supplementary Table S1).

### 2.3. SARS-CoV-2 antibody testing

As a part of the COKIBA study, which measured SARS-CoV-2 antibodies in SARS-CoV-2 hotspot and other regions of Bavaria in the summer of 2020 (10), Domspatzen students were invited to participate in antibody testing in September of 2020 using the following assays: the commercially available, licensed qualitative Elecsys Anti-SARS-CoV-2 (Roche Diagnostics, Rotkreuz, Switzerland; https://diagnostics.roche.com) and a validated and published in-house ELISA as previously described (10). In brief, the Elecsys Anti-SARS-CoV-2 assay does not discriminate between the antibody types IgA, IgM, and IgG and is based on a recombinant nucleocapsid (*N*) antigen and has a cutoff value of 1.0 (S/Co). The in-house ELISA is based on SARS-CoV-2 S-protein's receptor-binding domain, quantifies total IgG and has a cutoff value of 1.0 (S/Co). The detected reactivity correlates with the SARS-CoV-2 neutralization titer as described previously (10). All samples with S/Co <1.0 were considered negative.

### 2.4. Gargle-pool SARS-CoV-2 rRT-PCR testing

When STACADO started in September of 2020, no antigen tests were yet available on the market, nasal swabs were recommended by the WHO for rRT-PCR testing; and pooling of samples for PCR was discussed controversially only to be introduced later (13). rRT-PCR testing methods were developed in this study in two phases (Table 1): Phase 1 was from September 2020 to March 2021 and phase 2 was from March 2021 to March 2022. In this project, samples for rRT-PCR testing were collected by gargling and procedures were developed for a school setting. Gargling started with saline solution in the first phase of the study and was changed soon to distilled water due to better acceptance with students. In the second phase, gargling was performed with plain tap water. All solutions were tested successfully for concordance with PCR procedures.

**Table 1:** Comparison of gargle pool rRT-PCR testing in phase 1 (STACADO) and 2 (WICOVIR).

	Phase 1	Phase 2
Period	09/2020–02/2021	03/2021–12/2021
Laboratory	Synlab Weiden	WICOVIR laboratory at Regensburg Hospital
Gargle solution	Saline/distilled water	Tap water
Amount	10 ml	5–6 ml
Pool size	5 samples	Up to 30 samples
Pooling	In laboratory	At school
RNA isolation	Synlab procedure (patent pending)	Bead isolation
PCR method	Synlab procedure (patent pending)	2-gene PCR
Data transfer	Real time	Real time
Availability of test result	Same day evening	Same day noon

In phase 1 of the testing, rRT-PCR was performed in batches of 5 by Synlab, Weiden according to an in-lab procedure for which a patent is pending. In phase 2, rRT-PCR testing was performed as previously described in the WICOVIR study (9). Briefly, pools of up to 30 individuals (one class and attached staff members) were generated. RNA was isolated from each pool with Auto-Pure 96 system ((Hangzhou Allsheng Instruments, Shanghai, China) by using the MagnifiQ™ RNA buffer kit (A & A Biotechnology, Gdansk, Poland) with the capacity of isolation from 96 pools in 27 min. Then the RNA samples were run on the BIORAD Real-Time PCR System (CFX96; Bio-Rad, Hercules, California, USA) using triplex rRT-PCR to detect 2 genes of SARS-CoV-2 (ORF1b and N2 gene) and one human gene as an internal control (Rnase P gene).

### 2.5. Antigen testing

In March of 2021 schools in Bavaria were ordered by law to perform regular antigen tests on all students attending school. Test kits of numerous manufacturers were provided by the government for those mandatory tests. These antigen tests were thus performed outside of and in parallel to the study described here.

### 2.6. Isolation and quarantine

Persons who tested positive for SARS-CoV-2 were immediately asked to isolate themselves, and the local health authority was notified. Based on the date of a positive testing result (PCR-positive) or the beginning of symptoms the isolation time varied from 14 days in the beginning of the pandemic to five days depending on the national recommendations of the Robert Koch Institute as Germany's main institution for infection control and prevention, and legal obligations based on the German Infection Protection Act (Infektionsschutzgesetz, IfSG), or additional federal state laws, respectively. Susceptible contact persons were quarantined following the same recommendations/legal obligations. Isolation could take place in the boarding school or at home depending on the specific case and spatial capacity.

### 2.7. Anonymous survey on acceptance of measures

As part of the study a “citizen science” or rather “student science” approach was implemented in February of 2022. Students of the Domspatzen together with the principle investigators developed a questionnaire containing 5 major questions to assess the acceptance of COVID-19 related hygiene measures at school and the impact of these measures on their life in cooperation with the study team. This questionnaire was implemented in qnome.eu and distributed by the students to their peers and staff members to be answered anonymously via smart phone.

### 2.8. Statistical analysis

Only descriptive methods were used and thus, no statistical tests were applied.

## 3. Results

### 3.1. Singing scheme

**Figure 2:**
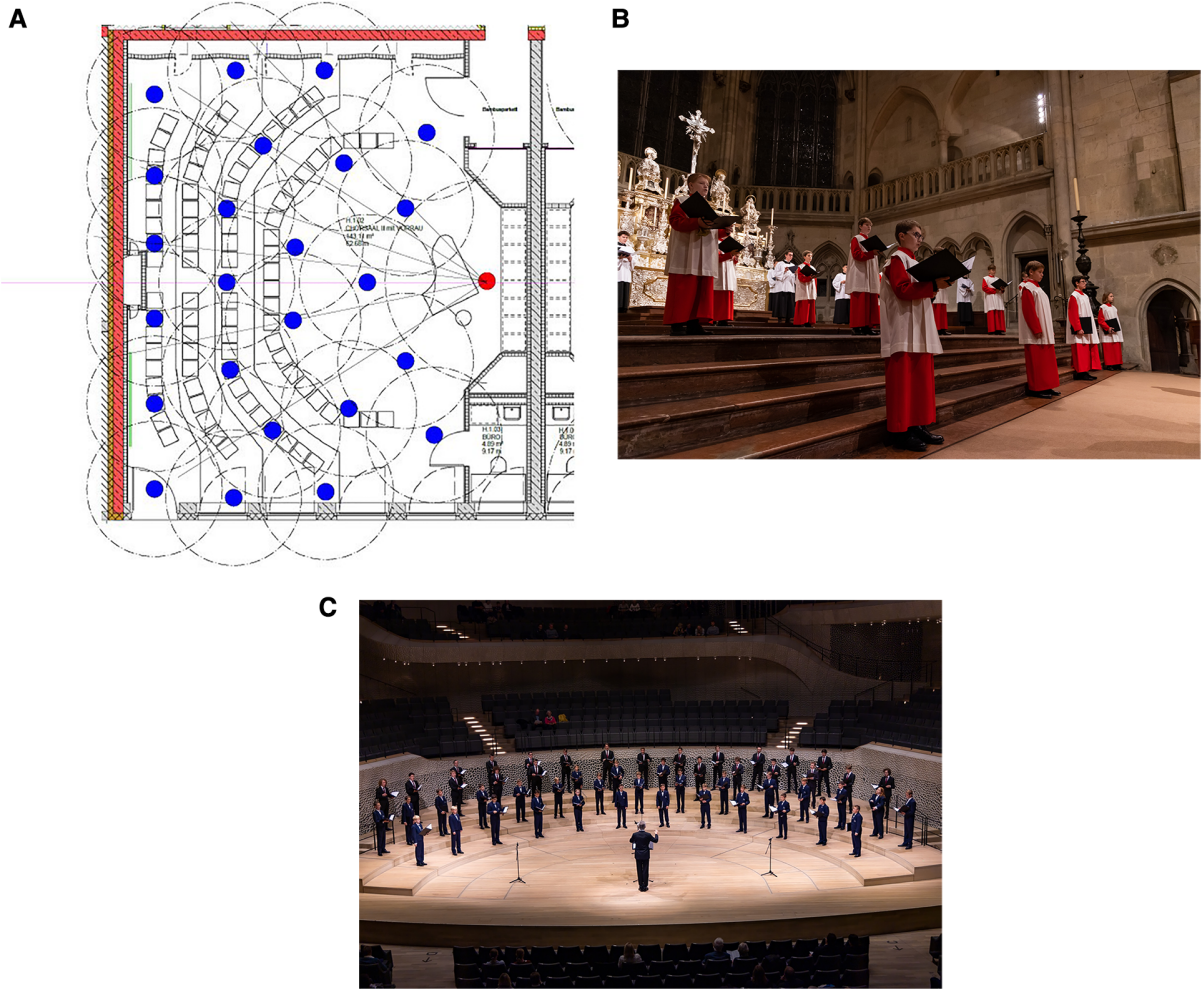
Singing scheme of domspatzen singers. (**A**) Positioning scheme for the rehearsal Hall of the Domspatzen boarding school (distance between singers: 3 meters). Alternative positions of choir master: red and white (piano) dot; fixed position of singers: blue dots; (**B**) positioning for singing at the mess at the Regensburg cathedral, December 2020; (**C**) positioning for a concert at Elbphilharmonie Hamburg in autumn of 2020.

Like all schools in Germany, the Domspatzen Boarding school was closed in lockdown very early and repeatedly in the course of the COVID-19 pandemic (Figure 1, Panel A). This was part of the national German strategy to fight the pandemic. NPI measures were implemented in schools in general but a specific set of procedures was introduced at Domspatzen, as it is a boarding and choir school (Figure 1, Panel B). Singing in any form was forbidden at times and concerts had to be canceled (Figure 1, Panel C). To make singing safe, an elaborate scheme to position singers was applied, distancing singers in their high-ceiling practice rooms (Figure 2A) as well as in the spacious Regensburg cathedral and in concert halls (Figures 2B,C, Table 2).

**Table 2:** Measures of rehearsal rooms and the regensburg cathedral.

	Floor space [m^2^]	Height [m]
Wolfgangsaal	354.97	5.52
Choir room Rädlinger	143.11	4.87
Choir room Giehl	143.08	4.87
Choir room 800	226.64	5.00
House chapel	292.11	5.50
Regensburg Cathedral	Total length inside: 85.40 Width inside: 34.80 m	Height of inner middle nave: 31.85

### 3.2. Testing regime

In the summer of 2020, we further hypothesized that establishing an elaborate test regime for SARS-CoV-2 allowing for an early isolation of positive members of the choir would additionally improve the safety at the school (Figure 1, Panel D), as infections could be detected before spreading. First, we investigated the status quo of infections in the Domspatzen school: In September of 2020, at the beginning of the schoolyear 2020/2021 and after the first COVID-19 wave in Germany, all students and staff were invited to participate in antibody testing which was performed according to the COKIBA protocol, investigating antibodies against N and S proteins. In total, 193 students (68%) and 78 staff members (57%) participated in the testing and 5 students (2.6%) and 2 staff members (2.6%) had antibodies in at least one of the two assays.

Next, we established the 1st phase of gargle pool rRT-PCR testing, also in September of 2020. Participation was voluntary and at the end of phase 2, 245 choir members participated in the STACADO testing. Initially, gargling was performed in the school (under supervision in corner-boxes of a big practicing hall) with 10 ml of isotonic saline solution/distilled water. Due to better acceptance by students, saline was soon replaced by distilled water and gargling was performed privately before school started in the morning. Twice a week (Monday and Thursday), individual samples were registered and collected in the school and transported to the test laboratory where samples were pooled (5 samples each) and analyzed according to an in-lab rRT-PCR protocol at Synlab laboratories in Weiden. With the partner MaganaMed GmbH we developed and established a software which retrieved laboratory results automatically from the partner lab via a secure connection and distributed results to the students based on an individual study ID.

In total, 2,148 samples were tested in 864 pools and 56 additional single rRT-PCR tests (for quality control of ambivalent primary results) from September 2020 to March 2021 (Figure 1, Panel D), and none of the gargle pool tests was positive (Figure 1, Panel E). However, one positive student was identified outside pool testing during that time: A 14-year-old choir boy was infected most likely by a family member directly after testing negatively in the STACADO pool rRT-PCR. Initially, he developed only mild symptoms not suggestive of COVID-19 and thus, he attended one choir practice and participated in the choral at Regensburg cathedral the following day. Another day later, he had full symptoms and was tested positively for SARS-CoV-2 by rRT-PCR. Due to the setup of regular testing in the choir, choir boys were tested repeatedly within a time span of 14 days after the event. None of the 50 exposed choir members became infected.

In the 2nd phase of testing (WICOVIR testing), which started in March of 2021 (Figure 1, Panel D), participants gargled with 5–6 ml of tap water twice a week at home. Now gargle samples were taken to the collection points in the school (usually in front of classrooms) and pooled at site by the students. Pools were registered and transported to the lab again twice per week, where they were analyzed. In the case of a positive pool, the school was informed, choir rehearsal in the afternoon canceled for that group and backup tubes of all participants of that specific pool were transported to the medical laboratory for individual rRT-PCR diagnostics. The individual test result was usually available within 6 h. In this 2nd phase, 4,416 samples were tested in 266 pools (and 26 single tests) from March 2021 until July 2021 (end of the school year) and 7,846 samples in 630 pools again from November 2021 until April 2022 (new school year). A total of 270 students (out of 282) and 60 staff members (out of 138) participated in the testing Three positive cases were identified by pool testing before the end of 2021. Two further positive cases occurred outside pool testing: One student decided to refrain from pool testing and perform only the mandatory antigen test. That test was negative but within hours thereafter he became symptomatic and the individual rRT-PCR test was positive. In the aftermath, a student with close contact also became positive (identified by pool testing early on) and thus, a direct infection cannot be excluded. Thus, 3 participants were tested positive by pool testing for the initial SARS- CoV-2 virus and alpha, beta and delta variants until the end of 2021. Except for the one case reported above when pool testing was not performed, no spreading of an infection in the school environment or in connection with singing practice was observed. Compared to the incidence proportions in the general population, we observed lower case numbers within the school until the end of 2021 (Figure 1, Panel E).

This changed with the occurrence of the Omicron variant in the beginning of 2022, when 78 individuals participating in pool testing were tested positive for the Omicron variant of SARS-CoV-2 within 3 months. Infections were so common that infection chains could not be determined any more but single infections may also have occurred in the school and during choir setting. Of note, the basic vaccination rate at Domspatzen (that is 2 vaccinations or one infection and one vaccination; mainly mRNA-based vaccines for children/adolescents or homologous/heterologous vaccination schemes [mRNA and/or vector based vaccines] following the national recommendations, which were adapted over time) was at 78% for choir members/students before the Omicron wave hit.

### 3.3. Survey on acceptance of hygiene measures

**Figure 3:**
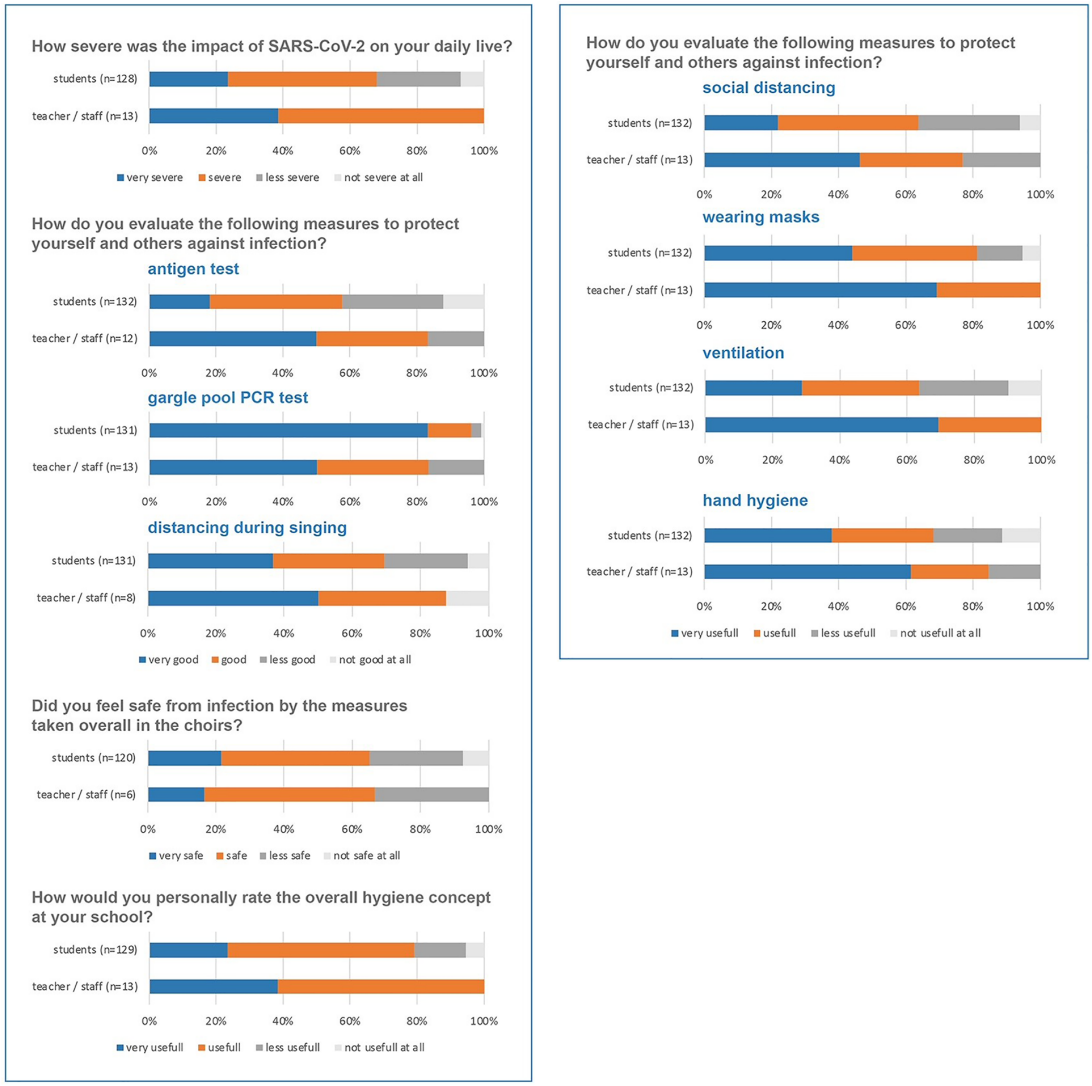
Results of questionnaire on the impact and acceptance of anti-SARS-CoV-2 measures.

To assess how hygiene measures were accepted by students and staff, an anonymous survey was performed in February of 2022, containing 5 major questions/domains. 134 students (51%) and 13 staff members (9%) filled in the complete questionnaire. A large majority of those that answered described the impact of the COVID-19 pandemic on their daily live as severe and very severe (71%) and 80% thought that the overall hygiene concept implemented by the school was useful to protect themselves and others from infection (Figure 3).

## 4. Discussion

We followed a world-famous youth choir for 2 years through the pandemic and recorded the measures taken to prevent corona-virus outbreaks in that setting, which included strict and extensive precaution measures during singing on top of general NPIs. As regular rRT-PCR based testing was performed almost continuously and prospectively since September 2020 and antibody screening before, this is a most comprehensive record of the pandemic in a choir up to date. Until the Omicron variant arrived, potential transmission of SARS-CoV-2 in the school was limited to only one case. During the omicron wave, infections were so common that tracing of exact routs of infection was not possible. However, no pattern associated with choir rehearsals or performances was observed, in the sense that an increase of transmissions associated with rehearsals or concerts was not conspicuous. Infection incidence at the school was never higher than in the comparable populations, suggesting that singing can be safe even during the COVID-19 pandemic.

Of those students and staff members who participated in the antibody testing in September of 2020, 2.6% were found positive. In comparison, a random sample of children from Regensburg from early summer of 2020 showed a share of antibody-positive individuals of 3% using exactly the same COKIBA test protocol as applied here (10). As the Domspatzen had at least 3 months more time to acquire SARS-CoV-2 infections but show a slightly lower rate of seroconversion, we conclude that Domspatzen members were not exposed to infection at a higher risk than the general population of that age, by singing with the elaborate hygiene measures in place. Also, residing at a boarding school did not increase the risk of infection for the singers. From September of 2020 on, we tested for SARS-CoV-2 infections prospectively by rRT-PCR. We did not find any evidence for an infection in the school from September of 2020 to March 2021. All students and staff members who tested positive for SARS-CoV-2 during that time acquired the virus outside the school. From March 2021 to the end of 2021, 3 participants were tested positive in the pool testing. One further infection occurred when a student decided to use antigen test instead of participating in pool PCR testing. The roommate was tested positive in the next pool rRT-PCR and was the only case of a suspected transmission within the school. When omicron became the dominating variant at the beginning of 2022, numerous students and staff members tested positive within the following months. However, the (estimated) incidence proportion was not different from other well tested populations (14) and furthermore, no super-spreading events in connection with choir practice or concerts were observed.

Measures to prevent transmission during singing such as virtual rehearsals/performances, face masks, reducing choir size, performing outside, optimized ventilation, physical distance, shorter rehearsals, regularly cleaning of surfaces and washing hands, or temperature screening were implemented at Domspatzen as scientifically suggested (15, 16). However, some of these measures are apt to compromise the quality of choir music and to impact significantly on the life of choir boys. This was assessed in a questionnaire developed with the students and filled out by the students. While impact on daily life and singing was rated as severe, most students still agreed with the usefulness of the measures. Interestingly, regular testing was viewed as the most important measure to increase safety in the school. We performed regular rRT-PCR testing of the choir boys for SARS-CoV-2 twice to three times weekly and before concerts while the choir introduced scientifically approved concepts of distancing during singing (12, 15). Whereas these measures are directed against outbreaks, single infections carried into the choir cannot be avoided, since that risk is linked to the overall pandemic situation in the population (Figure 1, Panel F). By testing Domspatzen members 2–3 time per week, not all infections could be detected in the asymptomatic state by gargle pool testing, which is to be expected due to the interval of testing and the rapid development of omicron infections because of the higher contagiousness of this variant. Indeed, the number of infections detected only in the symptomatic state per week is very comparable to other studies at the same time with the same method (14).

Our analysis is limited to one choir school and that limits generalizability of our results. When reaching out to other (semi) professional youth choirs in Europe, no similar activities were reported from any other site and data were not collected systematically. A further limitation of our study is, that data collection also within the Domspatzen setting was never complete as participation in the study was not compulsory. Also, the attitude towards measures such as testing may have been overly positive, as students were involved actively in the development of these methods and this may have biased their view on the usefulness of testing. On the other hand, similar results for the acceptance of the gargle pool testing were also achieved by questionnaire in a general school setting (9) and a hospital setting (14).

## 5. Conclusions

After following the Domspatzen choir closely over more than 2 years through the pandemic, implementing scientifically sound hygiene measures for singing and applying extensive monitoring of infections, we conclude that these measures may have countered a potentially excessive risk suggested to exist due to aerosol spreading of SARS-CoV-2 by singing. These measures are acceptable for choir members if this enables them to continue singing even in times of a pandemic.

## Data Availability

The raw data supporting the conclusions of this article will be made available by the authors, without undue reservation.
